# Vegetable Waste in the Retail Sector: Evaluation and Possibilities for Use in New Product Development

**DOI:** 10.3390/foods13182916

**Published:** 2024-09-14

**Authors:** Eduardo Galvão Leite das Chagas, Vitor Augusto dos Santos Garcia, Carla Alves Monaco Lourenço, Fernanda Maria Vanin, Cristiana Maria Pedroso Yoshida, Rosemary Aparecida de Carvalho

**Affiliations:** 1 Faculty of Animal Science and Food Engineering, University of São Paulo, Pirassununga 13635-900, SP, Brazil; eduardochagas@usp.br (E.G.L.d.C.); carla.monaco@usp.br (C.A.M.L.); fernanda.vanin@usp.br (F.M.V.); 2 Faculty of Agricultural Sciences, UNESP-São Paulo State University, Av. Universitária, Botucatu 18610-034, SP, Brazil; vitor.as.garcia@unesp.br; 3 Institute of Environmental, Chemical and Pharmaceutical Sciences, Federal University of São Paulo, Diadema 04021-001, SP, Brazil; cristiana.yoshida@unifesp.br

**Keywords:** food loss and waste, antioxidant compounds, retail waste, phenolic

## Abstract

Food waste is a significant concern when it comes to food safety. It is a well-known fact that fruit and vegetable wastage is high worldwide; however, quantitative data, especially on such waste in the retail sector, are limited. Wasted vegetables are sources of essential dietary compounds, benefiting from their revalorization. Studies related to the evaluation of the quantity and quality of these vegetables discarded in the retail sector can allow for the proposal of relevant alternatives for their use and the guarantee of food safety. This study aimed to assess wasted vegetables (beetroot, carrot, chayote, and cucumber) in the city of Pirassununga (Brazil) and characterize the purees obtained from these vegetable wastes. The purees were characterized in terms of microbiological aspects, color, proximal and mineral composition, phenolic concentration, and antioxidant activity. It was observed that 90% of the discarded vegetables were free from microbiological contamination and could be considered suitable for consumption according to the adopted classifications. Additionally, the purees had high nutritional levels, such as phenolics and fiber. Thus, considering the high levels of vegetable waste generated in the retail sector, and high value nutritional, vegetable waste could be viable in the development of new products, making it an important retail strategy for the circular economy.

## 1. Introduction

The continuous increase in the world’s population has raised concerns, especially regarding food security. According to UN estimates [1], by 2050, the world’s population will have increased by 18.01% compared to 2022. This means that food insecurity will also increase at an alarming rate, according to the FAO report [2], which states that in 2022, 29.6% of the world’s population was food insecure, with 11.3% affected at the most severe level.

In addition to food insecurity, another concern is the high levels of food loss and waste (FLW) worldwide. Food loss is defined as the food removed from the food chain at the harvesting, processing, or transportation stages, without being consumed [3]. Food losses represent around 13.8% of the total food produced in the world [3]. In turn, food waste, which is defined as the food removed from the food chain at the retail and consumption stages, corresponds to 17% of the total food available in the world [4]. These figures indicate that almost a third (30.8%) of the food available for consumption in the world is lost or wasted.

One of the major problems related to the evaluation, especially of food waste, is the lack of available data [5]. Bellemare et al. [6], in their study evaluating the methodologies for measuring food waste, added that even though there are data available in the literature, the differences in the methodologies used for quantifying losses have resulted in a high discrepancy in the levels, thus impairing comparison and compromising the final waste database.

According to the United Nations Environment Program (UNEP) [4], there is currently a significant shortage of data on food waste, exacerbated by the wide variation in the quantified data presented in a few existing studies. In its report, the UNEP [4] suggests that the actual levels of food waste may be up to double the levels reported in the literature. 

Information on food waste comprises information related to the disposal of food in the retail chain as well as in households (consumption). In the case of household waste, there is a high degree of uncertainty in the values owing to the difficulty of verifying the data reported by consumers in surveys [7].

In-depth studies related to food waste are fundamental to understanding and taking practical action to reduce waste. Although limited, some studies in the literature are important to guide and substantiate food waste data. For example, a study by Goodman-Smith et al. [8] evaluated food waste in New Zealand retail and reported that vegetables represent around 27% of the total volume of waste generated in this region. Caldeira et al. [9], in their study evaluating FLW in the European Union, indicated that, in general, vegetables contribute 46% of the total FLW. This means that 0.9 million tons of vegetables are wasted in retail alone, which corresponds to around 2% of all the vegetables produced in the world.

According to Oliveira, Lago, and Dal’ Magro [10], studies related to the circular economy report that foods classified as FLW (food loss and waste) often still have a high potential for reuse, recovery, and recycling and therefore could be fully exploited, avoiding waste to the maximum extent possible. 

Most studies related to vegetable waste focus on using it as a raw material for composting [11], as feed for animals such as dairy cattle [12], as biofertilizers [13], and in bioethanol production [14]. However, according to Sagar et al. [15], waste vegetables, even if classified as FLW, still have important concentrations of active and nutritional compounds (pigments, phenolic compounds, dietary fibers, organic acids, and minerals, among others) that can be beneficial to health. Transforming these wasted vegetables into purees is therefore relevant, as it extends their shelf life due to microbiological stability and enzyme deactivation [16]. In addition, purees made from waste vegetables are low-cost foods that can be used as ingredients in the development of new products such as pasta, cakes, and soups.

This study aimed to quantitatively assess vegetable waste and its potential for producing purees that could be used in the development of new products. The study examined microbiological safety and the composition of active compounds and antioxidants.

## 2. Material and Methods

### 2.1. Materials

The vegetables used in this study (beetroot, carrot, chayote, and cucumber) were discarded as “unsuitable” vegetables and donated by two supermarkets located in the city of Pirassununga (São Paulo, Brazil). The vegetables were defined based on the high quantity discarded and were collected by the researchers from the stores over a period of four months (at least twice a week), totaling 74.36 kg of carrots, 50.93 kg of beetroots, 61.47 kg of chayote, and 49.71 kg of cucumbers.

### 2.2. Classification and Determination of Vegetable Waste Percentage

After each collection, the vegetables were classified as “unsuitable” and “suitable”. Vegetables classified as “unsuitable” had microorganism growth on the surface, an altered texture (extremely soft surface), and an unpleasant smell. Vegetables that exhibited changes in shape and degree of ripeness but no microorganism growth and had a firm texture were classified as “suitable”. The mass of the vegetables was determined using a semi-analytical balance (Ohaus Corporation, ARD110, Parsippany, New Jersey, USA), and the percentages of “unsuitable” and “suitable” vegetables were determined by applying Equation (1) given below.

(1) $$\mathrm{USV}\;\mathrm{or}\;\mathrm{SV}=\frac{\mathrm{mass}\;\mathrm{of}\;\mathrm{USV}\;\mathrm{or}\;\mathrm{SV}\;(\mathrm{kg})}{\mathrm{total}\;\mathrm{mass}\;\mathrm{of}\;\mathrm{donated}\;\mathrm{vegetables}\;(\mathrm{kg})}\;\times \;100$$

where USV = unsuitable vegetables; SV = suitable vegetables.

### 2.3. Preparation of Vegetable Purees

The vegetables (beetroot, carrot, chayote, and cucumber) classified as suitable (only physical defects) were used and manually washed using running water and kept in a diluted sodium hypochlorite solution for the duration recommended by the manufacturer (8 mL per 1 L of water for 15 min; Triex, Sertãozinho, SP, Brazil). After this period, the vegetables were washed under running water for two minutes. To prepare the purees, the vegetables were peeled and cut manually (1 × 1 cm) using a vegetable chopper (Metal Ferreira, MFP-P, Itajobi, SP, Brazil) and steamed using a combi oven (Prática, C-MAX 3 gourmet, Pouso Alegre, MG, Brazil) at 98 °C for one hour (100% steam). After cooking, the vegetables were shredded using a cutter (Sammic, CKE-5, Linda-a-Velha, Lisboa, Portugal) for three minutes (medium speed).

The beetroot (BRP), carrot (CRP), chayote (CHP), and cucumber (CCP) purees were stored in metal trays and frozen at −22 °C for 24 h. One batch of frozen purees was homogenized and stored in airtight plastic bags (Zip Lock) at −22 °C (Brastemp, BVR28, São Paulo, SP, Brazil). To carry out the characterizations, the purees were thawed in a refrigerator (4 ± 2 °C) for 24 h and then stored at room temperature (25 ± 2 °C) until the time of analysis.

### 2.4. Microbiological Analysis of Vegetable Purees

The purees were analyzed for *Escherichia coli*, *Staphylococcus aureus*, and *Salmonella* sp. using commercial plates (Idexx, Compact Dry, Westbrook, ME, USA) as per the methodology proposed in the manufacturer’s manual [17]. For microbiological analyses, the puree samples (10 g) were dispersed in 90 mL of buffered peptone water (1%) and homogenized in a stomacher (Marconi, MA440, Piracicaba, SP, Brazil) for 60 s at room temperature (25 ± 2 °C). Aliquots (1 mL) of the undiluted puree were spread on each plate to analyze for the presence of *Escherichia coli* and *Staphylococcus aureus*. The plates were then incubated in a BOD (Marconi, MA 415, Piracicaba, SP, Brazil) at 35 ± 2 °C for 24 h. The results were expressed as colony-forming units (CFUs)/mL of the samples.

For *Salmonella* sp. analysis, samples of the purees dispersed in buffered peptone water were preincubated in a BOD (incubator Biochemical Oxygen Demand, Marconi, MA 415, Piracicaba, SP, Brazil) at 36 ± 2 °C for 16 h. After this period, aliquots (100 µL) of the purees were dispersed on a *Salmonella* sp. detection plate (Idexx, Compact Dry, Westbrook, ME, USA) and incubated in BOD at 42 ± 2 °C for 24 h. After incubation, the plates were evaluated and the results were expressed in CFUs/mL of the samples.

### 2.5. Color Parameters

Samples of the purees (10 g) were dispersed in Petri dishes, and their luminosity (L*), chroma a*, and chroma b* were determined using an AEROS non-contact benchtop spectrophotometer (HunterLab, Reston, VA, USA), calibrated with black and white standards. 

The results correspond to the arithmetic mean of 35 readings (7 random measurements of the surface area of the plate per second with a total analysis time of 5 s). The CIELab scale was used. The illuminant D65 was used for the analysis, and the observer angle was set at 10°. 

### 2.6. Proximate and Mineral Composition

The proximate composition of the purees was determined as per the methodology proposed by the Adolfo Lutz Institute [18]. First, samples of the purees were freeze-dried (FD 1.0-60E, Heto-Holten A/S, Allerød, Frederiksborg, Denmark). Their protein content was determined using the Kjeldahl method using a protein distiller (Tecnal, TE-036/1, Piracicaba, São Paulo, Brazil) and a nitrogen conversion factor of 6.25. The crude fiber content was determined through the Van Soest method, using ether (Synth, São Paulo, SP, Brazil) as a solvent. The lipid content was determined using a Soxhlet extractor (Quimis, M25008, Diadema, SP, Brazil), using 2 g of the dried puree sample and petroleum ether (Synth, São Paulo, SP, Brazil) as the solvent. The ash content was determined by incinerating the sample (5 g) in a muffle furnace (Quimis, 318M24, Diadema, SP, Brazil) at 550 ± 2 °C for 72 h.

The concentration of minerals in the purees was determined in the manner proposed by Nogueira and Souza [19]. The samples (0.5 g of freeze-dried puree) were subjected to nitric–perchloric digestion (nitric acid, 4 mL, 65%, *v*/*v*; perchloric acid, 2 mL, Synth, São Paulo, SP, Brazil). An atomic absorption spectrophotometer (Varian, Fast Sequential AA240FS, Santa Clara, CA, USA) was used to determine the levels of zinc, calcium, copper, magnesium, manganese, and iron. The potassium content was determined using a flame photometer (Micronal, B462, São Paulo, SP, Brazil). Sulfur and phosphorus concentrations were determined using a spectrophotometer (Femto, 600 Soft, São Paulo, SP, Brazil) at wavelengths of 420 and 660 nm, respectively. All the commercial salts used as standards were supplied by Sigma-Aldrich (Bellefonte, PA, USA). The mineral results were expressed as mg of mineral per g of vegetable puree. 

### 2.7. Total Phenolic Compounds and Antioxidant Potential

The active compounds in the samples were extracted using a solution of methanol (Merck, LiChrosolv^®^, Darmstadt, Germany) in water (70:30 *v*/*v*), as per the methodology proposed by Kamiloglu and Capanoglu [20], with modifications to the ratio of sample mass and solvent volume. Puree samples (BRP and CRP = 6 g; CHP and CCP = 12 g) were dispersed in 5 mL of solvent. The dispersions were kept in an ultrasonic bath (Ultronique, Eco-sonics Q5.9/25A, Indaiatuba, SP, Brazil) for 15 min under refrigeration at 4 ± 2 °C (water circulator, Marconi, MA 84/6, Piracicaba, SP, Brazil). The samples were then centrifuged at 2700× *g* for 10 min (Eppendorf centrifuge, 5430R, Hamburg, Germany) at 4 ± 2 °C. After centrifugation, the supernatant was removed. The extraction process was repeated three more times (final extract volume = 20 mL).

#### 2.7.1. Total Phenolic Concentration

The total phenolic concentration in the purees was determined as suggested by Singleton, Orthofer, and Lamuela-Raventós [21], using 0.5 mL aliquots of the extracts obtained and described above. The reagents, namely Folin–Ciocalteau (2.5 mL) and sodium carbonate solution (Synth, São Paulo, SP, Brazil) (7.5%; 2 mL), were added to the samples, and after homogenization, they were left to sit for two hours in the absence of light (25 ± 2 °C). After two hours, their absorbance was analyzed using a spectrophotometer (PerkinElmer, Lambda 35 UV-Vis, Shelton, CT, USA) at 740 nm. Gallic acid (Sigma-Aldrich, Bellefonte, PA, USA) was used as an external standard (2.5–5.3 mg/mL), and the results were expressed as mg of gallic acid/100 g of dry sample.

#### 2.7.2. Antioxidant Potential Using the Ferric Reducing Antioxidant Power (FRAP) Method

The antioxidant potential was determined using the FRAP method [22], by mixing 0.1 mL of the puree extract with a solution of FRAP reagent (2.9 mL). The solution was then homogenized in a vortex (IKA, Vortex 1 V1, Staufen, Baden-Württemberg, Germany) and incubated at 37 ± 2 °C (water bath, Marconi, MA 127, Piracicaba, Brazil) for 30 min. The absorbances of the samples were determined in a spectrophotometer (PerkinElmer, Lambda 35 UV-Vis, Shelton, CT, USA) at 593 nm. Trolox (Sigma-Aldrich, Bellefonte, PA, USA) was used as an external standard (2.5–25 µM), and the results were expressed as μmol of Trolox equivalent/100 g in a dry sample.

#### 2.7.3. Antioxidant Potential Using ABTS^•+^ Assay

The ABTS^•+^ methodology proposed by Re et al. [23] was used to determine the antioxidant potential in the samples. Aliquots (30 µL) of the extracts were added to an ethanolic solution of the ABTS^•+^ radical (3 mL, 2,2′-azino-bis(3-ethylbenzothiazoline-6-sulfonic) acid, Sigma-Aldrich, Bellefonte, PA, USA), homogenized in a vortex (IKA, Vortex 1 V1, Staufen, Baden-Württemberg, Germany), and left to sit for six minutes. The absorbances were then evaluated in a spectrophotometer (PerkinElmer, Lambda 35 UV-Vis, Shelton, CT, USA) at 734 nm, using Trolox as the external standard (100–2000 µM). The results were expressed as μMol Trolox equivalent/100 g dry sample.

#### 2.7.4. Antioxidant Potential Using Oxygen Radical Absorbance Capacity (ORAC)

The antioxidant potential of the samples was measured using the ORAC method, as described by Ou, Hampsch-Woodill, and Prior [24]. Microplates (96 cells, Greiner Bio-One, Kremsmünster, Austria) containing fluorescein solution (150 µL; 81 nM) and aliquots of the puree extracts (25 µL) were used for this purpose. The microplates were incubated at 37 ± 2 °C for 10 min in a spectrofluorimeter (BMG Labtech, FLUOstar OPTIMA, Offenburg, Germany). After incubation, with AAPH (25 µL; 2,2′-azobis(2-methylpropanimidamide) dihydrochloride; 152 mM; Sigma-Aldrich, Bellefonte, PA, USA) was added, and fluorescein decayed at 1 min intervals for 120 min (λ_excitation_: 485 nm; λ_emission_: 528 nm). Trolox was used as the external standard (8–96 µM), and the results were expressed as µmol of Trolox equivalent (TEq)/100 g of dry sample.

### 2.8. Statistical Analysis

All the analyses of the purees were carried out in triplicate, excluding the antioxidant potential and the concentration of total phenolics, which were extracted three times, and each extraction was analyzed in triplicate for each evaluated method, giving a total of nine samples for each puree. The results are expressed as mean ± standard deviation. Analysis of variance (ANOVA) was carried out with a 95% significance level (*p* < 0.05), and the difference between the means was determined through Duncan’s test using SAS software (SAS Inc., version 9.4, Cary, North Carolina, USA).

## 3. Results and Discussion

### 3.1. Collecting Vegetables and Quantifying Vegetable Waste

Table 1 shows examples of vegetables classified as “unsuitable” and “suitable.” Through visual observation, vegetables that showed high levels of deterioration (microbiological, changes in texture, etc.) were classified as “unsuitable”, while others that showed no relevant changes on their surface were classified as “suitable”.

**Figure 1:**
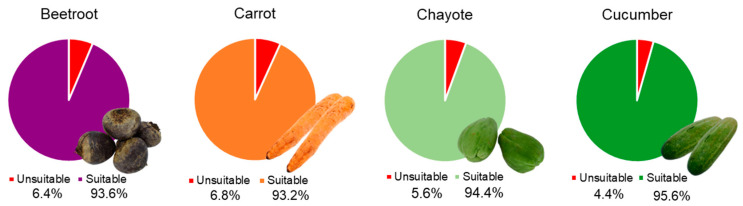
Percentage of vegetables wasted by supermarkets classified as “unsuitable” and “suitable” for consumption.

Regardless of variety, over 90% of the vegetables discarded by supermarkets (Figure 1) may, according to the classification used, be considered suitable for consumption. This indicates that a large volume of vegetables discarded by commercial establishments can be reused. Among the vegetables evaluated, cucumber had the highest percentage of being classified as suitable (Figure 1).

The possible reasons for supermarkets discarding vegetables could be related to the frequency at which the vegetables are removed from the shelves, the team responsible for this removal and management, the training of this team, the criteria established by the retail chains to ensure quality, the standards idealized and stipulated by consumers (no cuts, no breaks, standard color, etc.), and the average income of the consumers who frequently visit the establishments, among others. However, even after considering these hypotheses, the main causes of the high level of vegetable waste are complex and require new approaches to study.

Mattsson and Williams [25] reported that in supermarket retail chains in central Sweden, the possible causes of waste in the evaluated supermarkets were appearance, management format (or lack thereof), lack of quantitative disposal data, inadequate demand forecasting, and customer behavior and demands, among other factors. Based on the results of their study, the authors concluded that retail employees play a fundamental role in combating food waste—a fact that has been neglected in previous research. This still places them as the prime cause of waste, which, according to the authors, does not reflect the truth [25]. 

In addition to the above reasons, Shafiee-Jood and Cai [26] and De Moraes et al. [27] identified lack of management, lack of cooling systems, limits in the distribution system, and marketing strategies as factors contributing to high volumes of waste. Quantitative data on food waste in the literature reveal significant waste levels in retail chains. Mattsson and Williams [25] assessed fruit and vegetable waste in three retail chains in Sweden over the course of a year (December 2018 to November 2019) and reported that potatoes were the most wasted vegetable by mass (5600 kg wasted over the period of study), corresponding to 0.6% of all waste. Among the 19 most wasted foods (including fruits and vegetables) were carrots and cucumbers, each with 0.6% wastage (1300 kg and 900 kg, respectively) [25]. Mattsson and Williams [25] concluded that each type of fruit and vegetable presents a unique form of waste, and therefore there is no generic solution to end the general waste of these foods; tailored practices were required for each type of product. 

Goodman-Smith, Mirosa, and Skeaff [8] studied food waste in 16 retail stores in New Zealand cities (Auckland, Wellington, Christchurch, and Dunedin) and found that the greatest waste occurring in the retail sector was vegetables (27%), followed by bakery products (23%), meat and fish (19%), and fresh fruit (17%).

Lana and Moita [28] studied the visual quality of leafy vegetables and fresh herbs wasted in four retail stores in the Federal District of Brazil and reported significant variations in food wastage (from 8.7% to 97%). They observed that the lowest volumes of waste were spring onions, parsley, coriander, cabbage, leeks, and green leaf lettuce, while the highest volumes of waste (greater than 50%) were reported for romaine lettuce, escarole, chicory, mustard leaves, sage, and thyme. 

Data related to food waste are limited in the literature, with many studies scattered globally, making it challenging to evaluate and define strategies to reduce this waste, which implies economic losses. According to Liakou et al. [5], a significant amount of fruit and vegetables is wasted in retail, but information on wastage is limited. One hypothesis for the shallow levels of information contributing to difficulties in quantifying this waste is the inconsistency and ambiguity of the terms used, making it difficult to effectively compare the data [29]. 

Most studies on food waste tend to look only at the total value of waste, such as those by Mattsson and Williams [25] and Goodman-Smith, Mirosa, and Skeaff [8], and may or may not identify this waste as described above. However, studies aimed at evaluating the percentages of vegetables considered waste and whether or not they are suitable for consumption are currently limited, thus making it difficult to compare and understand how to treat these vegetables more appropriately. 

### 3.2. Microbiological Evaluation

Regardless of the type of vegetable, none of the purees showed growth of *Salmonella* sp., *Escherichia coli*, or *Staphylococcus aureus* (Table 2). These results indicate that the puree-based wasted vegetables are microbiologically safe and can be consumed or used to develop new products. 

In Brazil, the National Health Surveillance Agency (ANVISA) has stipulated, based on Normative Instruction No. 161/2022 [30], that vegetable purees must be free of *Salmonella* sp. and can have *Escherichia coli* up to a maximum limit of 10^2^ CFUs/g. Therefore, these vegetable purees, even if prepared from vegetables discarded as waste, are still suitable for consumption as they do not contain any undesirable microorganisms (*Salmonella* sp., *Escherichia coli*, and *Staphylococcus aureus*).

### 3.3. Color Parameters

One of the main visual characteristics of the purees is their color, which is considered an important parameter when choosing a vegetable for consumption. Visually, even after the process of their preparation (sanitizing, cutting, cooking, and grinding), the purees still retained the characteristic color of their respective vegetables in natura (Table 3).

The luminosity value of the beetroot puree was lower than that of the other purees (Table 4). This behavior may be due to the high concentration of dark pigments present in beetroots, such as betacyanin (intense purple hue) [31].

Carrot puree had the highest values with regard to the a* and b* parameters (Table 4), as it was characterized by an orange color, corroborating what is seen visually in Table 3. The a* and b* values of the beetroot puree reflected a tendency toward a purple color, as expected. This behavior is likely associated with the high presence of pigments (active compounds) such as carotenoids in carrots [32] and betalains in beetroots [33]. On the other hand, the chayote and cucumber purees showed negative results for the a* parameter and positive results for the b* parameter, reflecting a yellow-green color (Table 4).

Different results have been reported in the literature for the color parameters of vegetable purees. For example, Sonar et al. [34] and Patras et al. [35] observed lower results for carrot puree blanched at 98 °C (L* = 37.8; a* = 22.5; b* = 34.7) and crushed fresh carrot (L* = 31.46; a* = 14.61), respectively. Chandran et al. [33] reported values for beetroot puree boiled at 100 °C for 40 min (L* = 7.98; a* = 8.76; b* = 3.36). Shang et al. [36] evaluated chayote pulp (L* = 68.05; a* = −7.16; b* = 30.15), and Guiné, Henriques, and Barroca [37] reported lower values for fresh cucumber (L* = 64.68; a* = −10.15; b* = 25.07).

These differences may be associated with numerous factors, such as the heat treatment used; different pigment concentrations due to variations in the plant; and factors related to planting, such as ripening time, climatic conditions, and soil conditions.

### 3.4. Proximal and Mineral Composition

**Figure 2:**
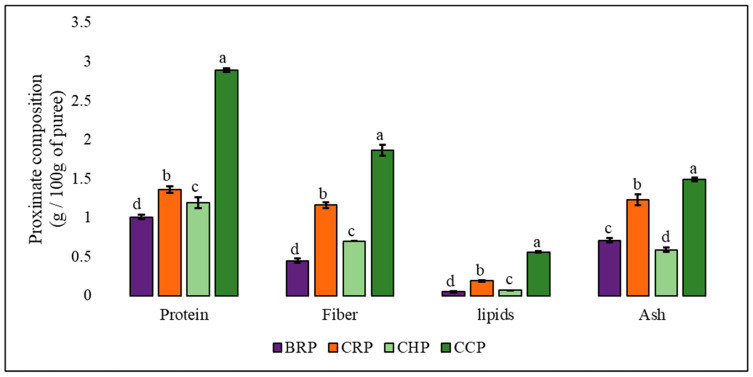
Analysis of the proximal composition of purees made from vegetables wasted in supermarkets. Note: BRP = beetroot puree; CRP = carrot puree; CHP = chayote puree; CCP = cucumber puree. Different letters in the same analysis indicate significant differences between the average values using Duncan’s test (*p* ≤ 0.05).

In general, all the purees showed significant concentrations of protein and fiber, as well as low levels of lipids (Figure 2). Among the purees, cucumber had significantly higher values for all the compounds (protein, fiber, lipids, and ash), possibly due to the presence of seeds. This behavior is related to the general physiology of vegetables, especially in relation to their morphological structure and composition [38].

The literature has revealed similar values of protein content in the studied vegetables, such as in the carrot puree with peel evaluated by Gomaa, Gomaa, and Abd El-All [39] (1.845 g protein/100 g sample); the carrot puree without peel evaluated by Prerana and Anupama [40] (0.65 g protein/100 g sample); the beetroot pulp (in natura) evaluated by Abdo et al. [41] (1.13 g of protein/100 g of samples); and the fresh cucumber pulp (*Cucumis sativus* L. cv. Bunex) characterized by Ruiz and Romero [42] (2.81 g of protein/100 g of sample). However, a reduced protein value was found in the fresh chayote pulp evaluated by Coronel et al. [43] (0.16 g of protein/100 g of sample).

In addition to protein, similar values have been reported in the literature for other compounds, such as in carrot puree (fiber = 4.57 g/100 g; lipids = 5.27 g/100 g) [39], beetroot pulp (fiber = 1.97 g/100 g; lipids = 0.15 g/100 g) [41], and chayote fruit (in natura) (fiber = 1.14 g/100 g; lipids = 0.13 g/100 g) [43].

In terms of the minerals present, it was observed that all the purees had iron, potassium, and zinc contents (Table 5), making these purees ideal for supplementing human diets. Minerals are essential for maintaining health, such as vitamin complexation [44], regulating human metabolism, and forming red blood cells through the concentration of iron [45]. 

Studies in the literature corroborate the results found here. For instance, Gomaa, Gomaa, and Abd El-All [39] reported similar levels of calcium (0.36 mg/g) and zinc (10.9 mg/kg) and higher levels of potassium (16.06 mg/g) and iron (32.4 mg/kg) in carrot pulp. Abdo et al. [41] characterized beetroot pulp (in natura) and reported higher mineral contents of iron (0.99 mg/g), phosphorus (0.41 mg/g), potassium (19.71 mg/g), calcium (1.55 mg/g), magnesium (1.16 mg/g), and zinc (1.77 × 10^−2^ mg/g).

Flick et al. [46] evaluated fresh chayote pulp and reported higher levels of iron (77.9 mg/kg), zinc (6.4 mg/kg), calcium (1.79 mg/g), potassium (12.87 mg/g), and magnesium (1.54 mg/g). Aghili et al. [47] reported similar concentrations of iron (3.1 mg/kg), zinc (2.1 mg/kg), potassium (2.31 mg/g), calcium (0.15 mg/g), magnesium (0.16 mg/g), and phosphorus (0.36 mg/g) when determining the mineral content of fresh cucumbers. 

Although some data in the literature report different values in relation to proximal and mineral composition, in general, the observed values are influenced by various factors, as observed in the study by Seljåsen et al. [48] evaluating the quality of carrots, where it was found that significant changes in proximate composition occur depending on the species, harvest time, soil composition, and climate, among others.

### 3.5. Total Phenolic Concentration

**Figure 3:**
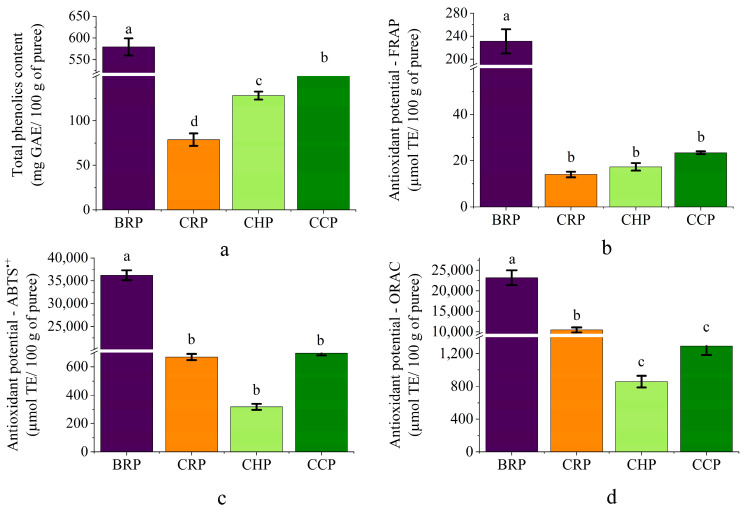
Concentration of total phenolics and antioxidant activity of purees of different vegetables wasted in supermarkets, with (**a**) concentration of total phenolics, and antioxidant activities by the methods of (**b**) FRAP (antioxidant activity by reduction of iron), (**c**) ABTS^•+^ (absorbance capacity of the ABTS radical), and (**d**) ORAC (absorbance capacity of oxygen radicals). Note: Different letters in the same analysis indicate significant differences between the average values using Duncan’s test (*p* ≤ 0.05).

The beetroot puree was found to have a significantly higher concentration of total phenolics than the other purees evaluated (Figure 3). According to Wruss et al. [49], beets have a high concentration of betalains (plant pigments), which, although not part of the phenolic compound class, have an impact on total phenolic concentrations. The authors found that the concentration of total phenolics in beetroot-based products is linearly dependent on the concentration of betalains. Thus, the higher concentration of total phenolics observed in beetroot puree may be associated with the high concentration of betalains present in the vegetable. 

The difference in phenolic compound content observed in different purees may be associated with the classes of phenolic compounds present in the vegetables (phenolic acids, hydroxycinnamic acids, flavonoids, and coumarins, among others), which can be altered or transformed throughout the vegetable production process (planting and harvesting), maturation, and when subjected to heat treatments [50].

Raczyk, Kruszewski, and Zachariasz [51], in their evaluation of different vegetable juices (tomato, carrot, and beetroot), observed that the concentration of total phenolics in beetroot juice was more than four times higher than the concentrations present in carrot and tomato juices.

Similarly, Stratil, Klejdus, and Kubáň [52] evaluated the concentration of total phenolics and antioxidant potential of different peeled and freeze-dried vegetables and reported higher concentrations of phenolic compounds in beetroot (2070 mg GAE/100 g of dry sample) compared to carrots and cucumbers (1450 and 1060 mg GAE/100 g of dry sample, respectively). The authors indicated that the difference might be due to various internal (vegetable color and water content) and external factors (climatic conditions, ripeness, post-harvest handling, and consumption time) [52].

Patras et al. [35] reported a similar concentration of total phenolics (102.80 mg GAE/100 g dry sample) in carrot puree, unlike Gomma, Gomma, and Abd El-All [39], who observed a lower concentration of total phenolics (27.67 mg GAE/100 g dry sample) in carrot puree. Riviello-Flores et al. [53], when evaluating chayote extracts and juices, found higher concentrations of total phenolics in the vegetable extract (525 mg GAE/100 g), and Sotiroudis et al. [54], when determining the concentration of phenolics in cucumber pulp (in natura), reported a lower concentration in this pulp (13.8 mg GAE/100 g of pulp).

### 3.6. Antioxidant Potential

As with the concentration of total phenolics, the beetroot puree had a significantly higher antioxidant potential than the other purees (Figure 3). This result may be due to the high concentrations of phenolic compounds found in the puree.

According to Cheynier [55], phenolics have antioxidant activity, which is important for benefiting health. Different studies in the literature have demonstrated the dependence of antioxidant potential on the concentration of total phenolics in vegetables. For example, Wruss et al. [49], in their study involving the characterization of different commercial products based on beetroot, indicated a linear dependence of antioxidant potential on the concentration of total phenolics. Stratil, Klejdus, and Kubáň [52] revealed that 26 types of freeze-dried vegetable pulp also had this dependence.

Thus, it can be seen that the puree with the highest concentration of total phenolics also had the highest antioxidant potential among the evaluated vegetables, i.e., in the order of beetroot > cucumber > chayote > carrot purees. In addition to phenolics, betalains, present as pigments in beetroot, may have also contributed to the increased concentration of antioxidant potential in the beetroot puree, since these compounds have a high antioxidant potential [56].

The carrot puree showed the lowest antioxidant potential of all the purees as evaluated using the FRAP method and the highest potential as evaluated using the ORAC method (with the exception of the beetroot puree). This behavior is possibly due to the apolar nature of the carotenoids present in carrots, which are not detected in the FRAP test [52], as this is a redox reduction test, which makes it difficult to reduce this compound [57].

On the other hand, using the ORAC method, it was possible to detect the pro-oxidant character of the carotenoids present in carrots because this method involves inhibiting the peroxyl radical induced by the thermal decomposition (37 °C) of the AAPH radical [58]. Thus, chelating compounds such as carotenoids acted more effectively in inhibiting oxidative species [59]. Therefore, due to their structure, carotenoids show a better antioxidant response to methods that use free radicals (such as ORAC) than methods that use oxidation–reduction reactions (e.g., FRAP), mainly because they are apolar molecules (making it difficult to donate or receive electrons).

Studies in the literature corroborate the results observed in this study. For example, Ali and Sharma [60], in their evaluation of the drying of diced carrots and beetroot, found that beetroot showed a higher antioxidant potential (DPPH) than carrot (43.12 and 34.44 mg DPPH/100 g, respectively).

Sun et al. [61], when evaluating carrot puree, reported a higher value using the ABTS^•+^ method (20.2 µmol Trolox equivalent/g dry mass), compared to other studies in the literature. Wruss et al. [49], evaluating beetroot varieties, determined a lower antioxidant potential for beetroot pulp (in natura) using the FRAP method (37.1 µM Trolox equivalent) and the ORAC method (37.9 µM Trolox equivalent). Chao et al. [62] found a higher antioxidant potential in fresh chayote pulp using the ORAC method (49.95 µmol Trolox equivalent/g dry mass).

Thus, it can be seen that the purees, even if produced from waste vegetables, still have an antioxidant potential similar to that of vegetables not classified as waste.

## 4. Conclusions

The results of this study show that a significant percentage of over 90% of the vegetables discarded by supermarkets can be considered suitable for consumption according to the classification used. The results are reinforced by microbiological analyses, which showed that the purees did not exhibit *Escherichia coli*, *Staphylococcus aureus*, or *Salmonella* sp. growth. The vegetables had a proximal composition and mineral content within the range reported in the literature, as well as relevant concentrations of phenolic compounds and antioxidant activity after processing. These results indicate the potential for the development of products from vegetable waste. 

## Figures and Tables

**Table 1:** Examples of the vegetables collected (beetroot, carrot, chayote, and cucumber) and classified as “unsuitable” and “suitable”.

Vegetable	Unsuitable	Suitable
Beetroot	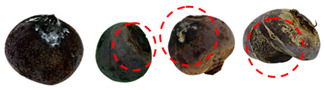	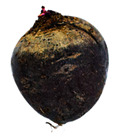
Carrot	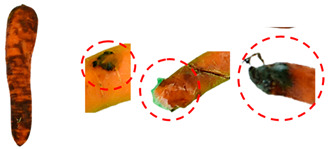	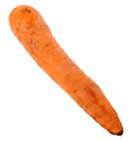
Chayote	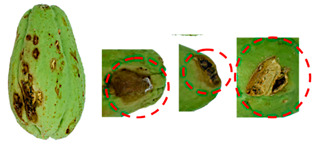	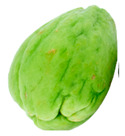
Cucumber	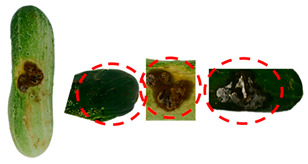	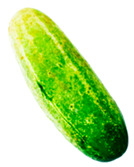

**Table 2:** Examples of microbiological tests (*Salmonella* sp., *Escherichia coli*, and *Staphylococcus aureus*) carried out on purees of vegetables wasted in supermarkets.

	Vegetable Puree			
**Type of microorganism**	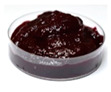	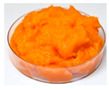	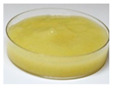	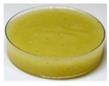
	BRP	CRP	CHP	CCP
*Salmonella* sp.	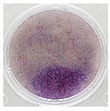	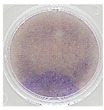	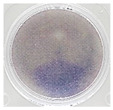	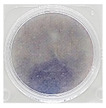
*Escherichia coli*	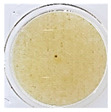	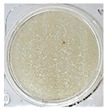	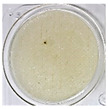	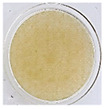
*Staphylococcus aureus*	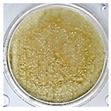	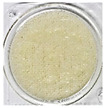	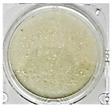	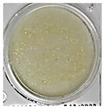

Note: BRP = beetroot puree; CRP = carrot puree; CHP = chayote puree; CCP = cucumber puree.

**Table 3:** Images of purees made from vegetables wasted in supermarkets.

Vegetable	BRP	CRP	CHP	CCP
Puree	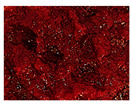	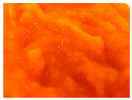	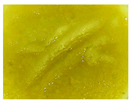	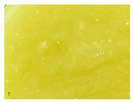

Note: BRP = beetroot puree; CRP = carrot puree; CHP = chayote puree; CCP = cucumber puree.

**Table 4:** Evaluation of the color parameters (luminosity—L*; chroma a* and chroma b*) of the purees produced using different vegetables (beetroot, carrot, chayote, and cucumber) wasted in supermarkets.

Puree	L*	a*	b*
BRP	14.84 ± 1.45 ^c^	9.02 ± 0.34 ^b^	2.29 ± 0.14 ^c^
CRP	48.09 ± 1.19 ^b^	33.51 ± 0.90 ^a^	51.38 ± 1.62 ^a^
CHP	58.39 ± 1.75 ^a^	−3.80 ± 0.10 ^d^	29.22 ± 1.13 ^b^
CCP	59.97 ± 1.08 ^a^	−0.90 ± 0.08 ^c^	29.32 ± 0.69 ^b^

Note: BRP = beetroot puree; CRP = carrot puree; CHP = chayote puree; CCP = cucumber puree. Different letters in the same column indicate statistically significant differences between the average values of the parameters determined using Duncan’s test (*p* ≤ 0.05).

**Table 5:** Mineral composition of purees made from different vegetables (BRP = beetroot puree; CRP = carrot puree; CHP = chayote puree; CCP = cucumber puree) wasted in supermarkets.

Mineral (mg/g of Puree)	Vegetable Puree			
	BRP	CRP	CHP	CCP
Nitrogen	1.58 ± 0.03 ^b^	2.06 ± 0.02 ^d^	1.87 ± 0.02 ^c^	4.46 ± 0.02 ^a^
Phosphorus	0.11 ± 0.02 ^c^	0.26 ± 0.04 ^bc^	0.22 ± 0.02 ^b^	0.57 ± 0.08 ^a^
Potassium	3.00 ± 0.25 ^b^	5.10 ± 0.10 ^ab^	2.98 ± 0.17 ^c^	7.13 ± 0.14 ^a^
Calcium	0.24 ± 0.05 ^b^	0.57 ± 0.02 ^a^	0.34 ± 0.00 ^b^	0.80 ± 0.01 ^a^
Magnesium	0.11 ± 0.00 ^b^	0.15 ± 0.02 ^c^	0.13 ± 0.00 ^b^	0.46 ± 0.01 ^a^
Sulfur	0.35 ± 0.03 ^c^	0.77 ± 0.01 ^b^	0.43 ± 0.02 ^c^	1.28 ± 0.12 ^a^
Copper	0.0012 ± 0.0001 ^ab^	0.0019 ± 0.0002 ^b^	0.0014 ± 0.0001 ^b^	0.0027 ± 0.0007 ^a^
Iron	0.0092 ± 0.0001 ^bc^	0.0148 ± 0.0006 ^c^	0.0116 ± 0.0002 ^b^	0.0216 ± 0.0008 ^a^
Zinc	0.0076 ± 0.0001 ^ab^	0.0119 ± 0.0007 ^b^	0.0090 ± 0.0001 ^b^	0.0164 ± 0.0003 ^a^

Note: Different letters in the same column indicate significant differences between the average values using Duncan’s test (*p* ≤ 0.05).

## Data Availability

The original contributions presented in the study are included in the article, further inquiries can be directed to the corresponding author.
